# Magnesium status modulating the effect of serum vitamin D levels on retinopathy: National Health and Nutrition Examination Survey 2005 to 2008

**DOI:** 10.3389/fnut.2024.1408497

**Published:** 2024-06-04

**Authors:** Lei Xu, Penghua Yuan, Wanrong Liu, Linlin Liu, Xiongfeng Li, Lianfeng Xie

**Affiliations:** ^1^Department of Ophthalmology, First Affiliated Hospital of Gannan Medical University, Ganzhou, Jiangxi, China; ^2^Department of Ophthalmology, Yudu County People’s Hospital, Ganzhou, Jiangxi, China

**Keywords:** vitamin D, magnesium, retinopathy, moderating effect, magnesium depletion score

## Abstract

**Aim:**

Magnesium levels may influence the effect of vitamin D levels on the body. This study aimed to assess the combined effect of magnesium status as reflected by magnesium depletion score (MDS) and vitamin D status on the risk of retinopathy.

**Methods:**

This cross-sectional study included participants aged 40 years and older with complete information on vitamin D, MDS, and retinopathy assessment from the 2005–2008 National Health and Nutrition Examination Survey (NHANES). Logistic regression analysis was utilized to analyze the relationship of MDS and vitamin D with retinopathy and expressed as odds ratio (OR) and 95% confidence interval (CI).

**Results:**

Of these 4,953 participants included, 602 (9.53%) participants had retinopathy. Serum vitamin D levels ≤30 nmol/L (vs. >30 nmol/L) (OR = 1.38, 95%CI: 1.05–1.81) and MDS >2 points (vs. ≤2 points) (OR = 1.47, 95%CI: 1.01–2.16) were associated with higher odds of retinopathy. There was an interaction between MDS and vitamin D on the increased odds of retinopathy (OR = 2.29, 95%CI: 1.12–4.68, *P*_interaction_ = 0.025). In different MDS groups, serum vitamin D levels ≤30 nmol/L increased the odds of retinopathy only in the MDS >2 group (OR = 2.90, 95%CI: 1.16–7.24), but not in the MDS ≤2 group (*p* = 0.293). Subgroups analyses demonstrated that the interaction between MDS and serum vitamin D on retinopathy was observed in males (OR = 6.88, 95%CI: 1.41–33.66, *P*_interaction_ = 0.019), people with diabetes (OR = 3.43, 95%CI: 1.78–6.63, *P*_interaction_ < 0.001), and people with body mass index (BMI) ≥25 kg/m^2^ (OR = 2.46, 95%CI: 1.11–5.44, *P*_interaction_ = 0.028).

**Conclusion:**

Magnesium plays a moderating role in the relationship between serum vitamin D and retinopathy. The protective effect of vitamin D against retinopathy was primarily present among those with inadequate magnesium levels.

## Introduction

Retinopathy is one of the major diseases that cause visual impairment and blindness, among which diabetic retinopathy is the leading cause of blindness in middle-aged and older adults worldwide (1). There is also a 6.7 to 18% prevalence of retinopathy in the population without diabetes, which may be related to advanced age and hypertension (2). Identifying modifiable factors that affect the risk of developing retinopathy is beneficial for disease prevention and reducing the burden of disease.

The retina is susceptible to oxidative stress (3). Vitamin D has been reported to prevent oxidative stress and inflammation in human retinal cells (4). Vitamin D may play a protective role in the retina through antioxidant, anti-inflammatory, anti-angiogenic, and immunomodulatory mechanisms (4, 5). In addition, diabetes is one of the major risk factors for retinopathy, and vitamin D may protect the retina by improving insulin sensitivity and decreasing insulin resistance (6). Magnesium is an essential nutrient that plays an important role in the regulation of blood pressure, glucose metabolism, vascular tone (7, 8), and it is involved in the synthesis and metabolism of vitamin D (9). Several studies have found that the effects of vitamin D on the body may vary depending on magnesium levels (10, 11). For example, the relationship between serum 25-hydroxyvitamin D [25(OH)D] and the risk of death may be altered by the level of magnesium intake, and this negative correlation was found mainly in populations with higher magnesium intake (10). However, the joint effect of serum magnesium levels and vitamin levels on retinopathy is unclear. Furthermore, blood magnesium accounts for approximately 1% of whole-body magnesium, and although serum magnesium measurements can be used for the medical diagnosis of clinically severe magnesium deficiency, they do not reliably represent whole-body magnesium status (12, 13). Since magnesium reabsorption in the kidney plays a crucial role in maintaining magnesium homeostasis (14), the magnesium depletion score (MDS) has been proposed as a new marker of magnesium status (15). MDS has been reported to be associated with self-reported risk of diabetic retinopathy (16).

Thus, this study aimed to assess the combined effect of magnesium status as reflected by MDS and vitamin D status on the risk of retinopathy in the middle-aged and elderly population, and to provide certain references for the prevention and management of retinopathy.

## Methods

### Study design and participants

The National Health and Nutrition Examination Survey (NHANES) dataset from 2005 to 2008 was used for this cross-sectional study. NHANES is an ongoing cross-sectional survey of health and nutrition of the United States noninstitutionalized population conducted by the National Center for Health Statistics (NCHS) of the Centers for Disease Control and Prevention (CDC).^1^ The NHANES survey utilizes a complex multi-stage probability sampling design with a two-year survey cycle. NHANES collects data through interviews and physical examinations, including demographic, dietary, socioeconomic, and health-related data, as well as medical, physiologic measurements, and laboratory test data. This study was based on two NHANES survey cycles, 2005–2006 and 2007–2008, because only these two cycles included full information on retinopathy based on retinal imaging exam. Participants were included according to the following criteria: (1) aged ≥40 years old; (2) with retinopathy assessment using retinal imaging; (3) with measurement of serum vitamin D; and (4) with complete information to calculate MDS. The excluded criteria were as follows: (1) with renal failure [estimated glomerular filtration rate (eGFR) <15 mL/(min·1.73 m^2^)] (17); (2) using anti-angiogenic ophthalmic agents, ophthalmic steroids; and (3) with missing key covariates. Only participants in NHANES aged 40 years and older were included in this study because two-field, non-mydriatic retinal photography was performed only on this age group. The NCHS Research Ethics Review Board approved all NHANES protocols and each participant provided written informed consent.

### Assessment of retinopathy

Non-mydriatic digital images of the retina were captured from participants aged ≥40 years using the Canon CR6-45NM ophthalmic digital imaging system and Canon EOS 10D digital camera (Canon USA Inc., One Canon Park, Melville, New York). Two digital images were taken of each eye of the participants in an almost completely dark room, with the first image centered on the macula and the second on the optic nerve. Digital images were evaluated by graders at the University of Wisconsin according to a modified Airlie House classification system (18). Retinopathy severity was graded according to the Early Treatment Diabetic Retinopathy Study (ETDRS) grading scale (18). Participants with levels ≥14 were considered to have retinopathy according to the eye with the worse retinopathy level. The detailed assessment process is described in the NHANES database (19).

### Assessment of MDS and vitamin D levels

The MDS was used to assess the total body magnesium status and was calculated using 4 factors: (1) diuretic use (current use for 1 point), (2) proton pump inhibitor use (current use for 1 point), (3) kidney function [60 mL/(min · 1.73 m^2^) ≤ eGFR <90 mL/(min · 1.73 m^2^) for 1 point; eGFR <60 mL/(min · 1.73 m2) for 2 points], and (4) alcohol consumption (heavy drinker for 1 point) (15). Heavy drinkers were defined as >1 drink/day for women and > 2 drinks/day for men. In this study, MDS was categorized as ≤2 and > 2.

Serum vitamin D levels were obtained directly from NHANES records based on laboratory test data. Severe vitamin D deficiency with a serum 25(OH)D concentration below <30 nmol/L greatly increases the risk of mortality and many other diseases (20). In this study, vitamin D levels were categorized as ≤30 nmol/L and > 30 nmol/L.

### Covariates

Participants’ data were collected including age, gender, race, education, marital status, family poverty-to-income ratio (PIR), physical activity, smoking, diabetes, hypertension, dyslipidemia, cardiovascular disease (CVD), chronic kidney disease (CKD), dialysis, body mass index (BMI), time of venipuncture (morning, afternoon, evening), season of sample collection (November 1 through April, May 1 through October), vitamin A intake, vitamin D intake, Healthy Eating Index-2015 (HEI-2015), magnesium intake, and total energy intake. CVD includes angina, heart failure, heart attack, coronary heart disease, stroke, and congestive heart failure, and CVD was determined through self-report or the use of CVD medications. Diabetes (21), hypertension (22), and dyslipidemia (23) were identified in the basis of self-report or corresponding biochemical diagnostic indicators or appropriate medications. CKD was identified by a urine albumin to creatinine ratio (UACR) ≥ 30 mg/g or an eGFR ≤60 mL/min/m^2^ (24). Vitamin D intake includes dietary and supplemental intake, and vitamin D intake was categorized as adequate, inadequate, and unknown according to the Dietary Reference Intakes (25).

### Statistical analysis

Descriptive statistical analysis was performed in participants with and without retinopathy. Continuous data were described as mean and standard error (S.E.), and independent samples *t*-test was utilized to compare differences between the two groups. Categorical data were presented as frequency and percentage, and chi-square test or rank-sum test was used to compare differences between the two groups.

Variables with more missing values (e.g., physical activity, vitamin D intake, dialysis) were categorized as unknown, and variables with fewer missing values (<10%) were interpolated for missing values by the random forest multiple interpolation method using the “miceforest” package of the Python software. Difference analysis before and after missing value interpolation was performed (Supplementary Table S1). Weighted univariable logistic regression analysis was used to screen for covariates related to retinopathy (Supplementary Table S2). Weighted univariable and multivariable logistic regression analyses were utilized to assess the relationship of MDS and vitamin D with retinopathy: crude model was a univariable analysis; model 1 was a multivariable analysis that adjusted for age, gender, race, education, and PIR; model 2 was a multivariable analysis that adjusted for age, gender, race, education, PIR, diabetes, hypertension, CVD, CKD, dialysis, BMI, time of venipuncture, and vitamin D intake. The results were expressed as odds ratio (OR) and 95% confidence interval (CI).

The moderating effect of MDS on the relationship between serum vitamin D and retinopathy was analyzed. Crude model* included variables MDS, serum vitamin D, and interaction term “MDS × serum vitamin D.” Model 3 adjusted for age, gender, race, education, and PIR based on crude model*. Model 4 adjusted for age, gender, race, education, PIR, diabetes, hypertension, CVD, CKD, dialysis, BMI, time of venipuncture, and vitamin D intake based on crude model*. The interaction term “MDS × serum vitamin D” was used to assess the moderating effect of MDS on the relationship between serum vitamin D and retinopathy. In addition, the effect of the association between serum vitamin D and retinopathy was stratified in two groups of MDS (MDS > 2 and MDS ≤ 2). Subgroups analyses were performed based on gender, age, diabetes, and BMI.

Data cleaning and processing of missing values were performed using Python 3.9 (Python Software Foundation, Delaware, United States), and statistical analysis was performed using SAS 9.4 (SAS Institute Inc., Cary, NC, United States). All statistical tests were performed using two-sided tests, and a *p*-value of <0.05 was considered statistically significant.

## Results

### Characteristics of participants

**Figure 1:**
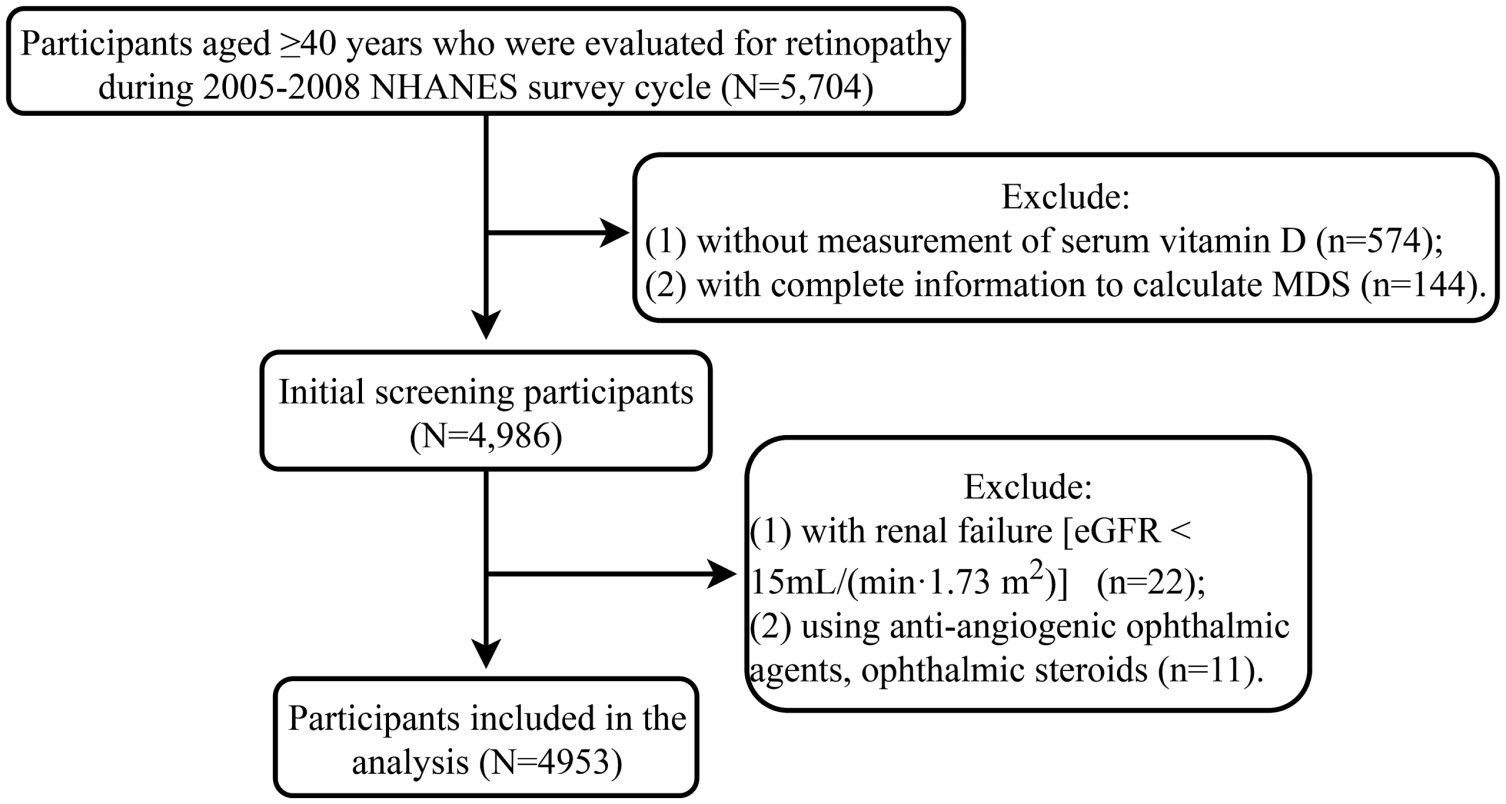
Flow chart of the study population. MDS, magnesium depletion score; eGFR, estimated glomerular filtration rate; NHANES, the National Health and Nutrition Examination Survey database.

During 2005–2008 NHANES survey cycle, 5,704 participants aged ≥40 years who were evaluated for retinopathy were selected. A total of 651 participants were excluded and 4,953 participants were included in the analysis (Figure 1). The characteristics of 4,953 participants were shown in Table 1. The mean age of the participants was 56.37 (0.39) years, of which 2,473 (35.82%) were ≥ 60 years old. There were 2,471 (52.48%) females and 2,723 (78.22%) non-Hispanic Whites. The mean BMI was 29.09 (0.15) kg/m^2^, and 3,677 (72.13%) participants had a BMI ≥25 kg/m^2^. The mean serum vitamin D level was 64.41 (0.76) nmol/L, and 557 (6.90%) participants had vitamin D levels ≤30 nmol/L. The mean MDS was 0.99 (0.03) points, and 432 (6.88%) participants had MDS >2 points. There were 602 (9.53%) participants with retinopathy and 4,351 (90.47%) participants without retinopathy.

**Table 1:** Characteristics of participants with and without retinopathy.

Variables	Total (*n* = 4,953)	Non-retinopathy (*n* = 4,351)	Retinopathy (*n* = 602)	*p*
Serum Vitamin D, *n* (%)				<0.001
>30 nmol/L	4,396 (93.10)	3,886 (90.85)	510 (9.15)	
≤30 nmol/L	557 (6.90)	465 (85.30)	92 (14.70)	
MDS, *n* (%)				<0.001
≤2	4,521 (93.12)	3,996 (90.89)	525 (9.11)	
>2	432 (6.88)	355 (84.73)	77 (15.27)	
Age, *n* (%)				<0.001
<60 years	2,480 (64.18)	2,244 (92.05)	236 (7.95)	
≥60 years	2,473 (35.82)	2,107 (87.63)	366 (12.37)	
Gender, *n* (%)				<0.001
Male	2,482 (47.52)	2,142 (88.66)	340 (11.34)	
Female	2,471 (52.48)	2,209 (92.10)	262 (7.90)	
Race, *n* (%)				<0.001
Non-Hispanic White	2,723 (78.22)	2,467 (91.55)	256 (8.45)	
Non-Hispanic Black	951 (8.86)	780 (84.57)	171 (15.43)	
Others	1,279 (12.93)	1,104 (87.95)	175 (12.05)	
Education, *n* (%)				<0.001
Less than high school	1,426 (17.36)	1,198 (86.64)	228 (13.36)	
More than high school	3,527 (82.64)	3,153 (91.27)	374 (8.73)	
Marital status, *n* (%)				0.559
Married	3,007 (65.60)	2,638 (90.45)	369 (9.55)	
Never married	337 (6.11)	297 (88.64)	40 (11.36)	
Others	1,609 (28.29)	1,416 (90.89)	193 (9.11)	
PIR, *n* (%)				<0.001
≤1.3	1,221 (14.50)	1,063 (89.57)	158 (10.43)	
1.3–3.5	1906 (33.97)	1,633 (87.32)	273 (12.68)	
>3.5	1826 (51.53)	1,655 (92.79)	171 (7.21)	
Physical activity, *n* (%)				0.002
<450 met*minutes/week	703 (15.22)	619 (91.26)	84 (8.74)	
≥450 met*minutes/week	2,431 (53.59)	2,188 (91.80)	243 (8.20)	
Unknown	1819 (31.19)	1,544 (87.78)	275 (12.22)	
Smoke, *n* (%)				0.707
No	2,362 (48.61)	2075 (90.66)	287 (9.34)	
Yes	2,591 (51.39)	2,276 (90.28)	315 (9.72)	
Diabetes, *n* (%)				<0.001
No	3,821 (83.37)	3,538 (93.44)	283 (6.56)	
Yes	1,132 (16.63)	813 (75.56)	319 (24.44)	
Hypertension, *n* (%)				<0.001
No	2023 (46.62)	1865 (92.83)	158 (7.17)	
Yes	2,930 (53.38)	2,486 (88.40)	444 (11.60)	
Dyslipidemia, *n* (%)				0.714
No	903 (18.38)	808 (90.93)	95 (9.07)	
Yes	4,050 (81.62)	3,543 (90.36)	507 (9.64)	
CVD, *n* (%)				<0.001
No	3,517 (75.96)	3,174 (92.29)	343 (7.71)	
Yes	1,436 (24.04)	1,177 (84.71)	259 (15.29)	
CKD, *n* (%)				<0.001
No	4,025 (86.29)	3,635 (91.85)	390 (8.15)	
Yes	928 (13.71)	716 (81.73)	212 (18.27)	
Dialysis, *n* (%)				<0.001
No	142 (2.19)	112 (83.51)	30 (16.49)	
Yes	9 (0.07)	3 (36.98)	6 (63.02)	
Unknown	4,802 (97.74)	4,236 (90.66)	566 (9.34)	
BMI, *n* (%)				<0.001
BMI < 25 kg/m^2^	1,276 (27.87)	1,171 (93.38)	105 (6.62)	
BMI ≥ 25 kg/m^2^	3,677 (72.13)	3,180 (89.34)	497 (10.66)	
Time of venipuncture, *n* (%)				0.025
Morning	2,394 (48.60)	2077 (89.24)	317 (10.76)	
Afternoon	1892 (35.63)	1,670 (90.90)	222 (9.10)	
Evening	667 (15.77)	604 (93.24)	63 (6.76)	
Season of sample collection, *n* (%)				0.352
November 1 through April	2,128 (36.55)	1857 (89.90)	271 (10.10)	
May 1 through October	2,825 (63.45)	2,494 (90.79)	331 (9.21)	
Vitamin A intake, mcg, Mean (S.E)	637.52 (11.63)	636.71 (11.31)	645.17 (28.78)	0.749
Vitamin D intake, *n* (%)				0.214
Adequate	477 (10.67)	430 (92.47)	47 (7.53)	
Inadequate	2,132 (35.91)	1868 (89.82)	264 (10.18)	
Unknown	2,344 (53.42)	2053 (90.50)	291 (9.50)	
HEI-2015, Mean (S.E)	51.40 (0.43)	51.51 (0.45)	50.43 (0.68)	0.153
Magnesium intake, mg, Mean (S.E)	320.44 (6.81)	321.05 (7.18)	314.59 (12.91)	0.642
Total energy, kcal, Mean (S.E)	2097.52 (21.29)	2098.16 (19.48)	2091.44 (71.57)	0.920

MDS, magnesium depletion score; PIR, family poverty-to-income ratio; CVD, cardiovascular disease; CKD, chronic kidney disease; BMI, body mass index; HEI-2015, Healthy Eating Index-2015.

### Association of MDS and vitamin D with retinopathy

Table 2 lists the association of MDS and vitamin D with retinopathy. Serum vitamin D levels ≤30 nmol/L (vs. >30 nmol/L) increased the odds of retinopathy in univariable analysis (OR = 1.71, 95%CI: 1.30–2.25) and multivariable analysis [model 1: (OR = 1.38, 95%CI: 1.05–1.81); model 2: (OR = 1.37, 95%CI: 1.01–1.87)]. MDS >2 points (vs. ≤2 points) was associated with higher odds of retinopathy in univariable analysis (OR = 1.80, 95%CI: 1.25–2.60). After adjusting for age, gender, race, education, and PIR, MDS >2 points (vs. ≤2 points) still increased the odds of retinopathy (OR = 1.47, 95%CI: 1.01–2.16), but not in analysis adjusted for all confounders (*p* = 0.482).

**Table 2:** Association of MDS and vitamin D with retinopathy analyzed by logistic regression analysis.

Variables	Crude Model		Model 1		Model 2	
	OR (95% CI)	*p*	OR (95% CI)	*p*	OR (95% CI)	*p*
Serum vitamin D						
>30 nmol/L	Ref		Ref		Ref	
≤30 nmol/L	1.71 (1.30–2.25)	<0.001	1.38 (1.05–1.81)	0.023	1.37 (1.01–1.87)	0.046
MDS						
≤2	Ref		Ref		Ref	
>2	1.80 (1.25–2.60)	0.003	1.47 (1.01–2.16)	0.049	0.86 (0.57–1.32)	0.482

MDS, magnesium depletion score; OR, odds ratio; CI, confidence interval; Ref, reference; Crude model, univariable analysis. Model 1, multivariable analysis that adjusted for age, gender, race, education, and PIR; Model 2, multivariable analysis that adjusted for age, gender, race, education, PIR, diabetes, hypertension, CVD, CKD, dialysis, BMI, time of venipuncture, and vitamin D intake.

### Moderating effect of MDS on the relationship between serum vitamin D and retinopathy

**Figure 2:**
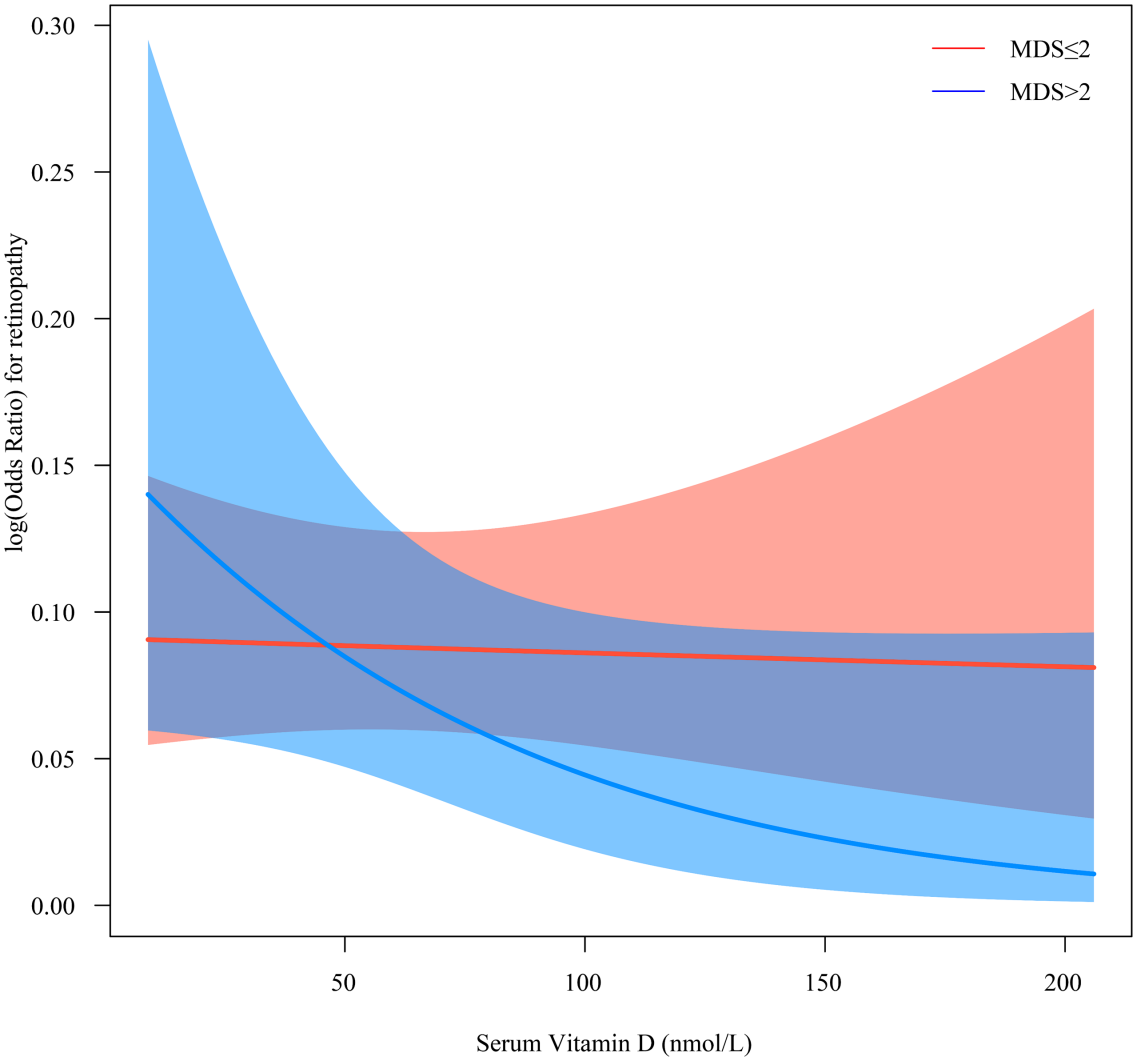
The interaction between MDS and serum vitamin D on retinopathy. MDS, magnesium depletion score.

Table 3 shows the effect of interaction term “MDS × vitamin D” on retinopathy. There was an interaction between MDS and vitamin D on the increased odds of retinopathy [crude model*: (OR = 2.33, 95%CI: 1.17–4.64), *P*_interaction_ = 0.018; model 3: (OR = 2.76, 95%CI: 1.40–5.45), *P*_interaction_ = 0.005; model 4: (OR = 2.29, 95%CI: 1.12–4.68), *P*_interaction_ = 0.025]. Figure 2 shows the interaction between MDS and serum vitamin D on retinopathy. The risk of retinopathy showed a relatively smooth trend with decreasing serum vitamin D levels in the MDS ≤2 group, whereas the risk of retinopathy showed a rapid increase with decreasing serum vitamin D levels in the MDS >2 group. These results suggest that MDS plays a moderating role in the relationship between serum vitamin D and retinopathy.

**Table 3:** Interaction between MDS and serum vitamin D on retinopathy analyzed by logistic regression analysis.

Variables	Crude Model*		Model 3		Model 4	
	OR (95% CI)	*p*	OR (95% CI)	*p*	OR (95% CI)	*p*
Serum vitamin D	1.47 (1.05–2.07)	0.026	1.16 (0.83–1.62)	0.380	1.06 (0.72–1.56)	0.767
MDS	1.56 (1.00–2.44)	0.052	1.26 (0.79–2.01)	0.317	0.78 (0.47–1.28)	0.312
MDS × serum vitamin D	2.33 (1.17–4.64)	0.018	2.76 (1.40–5.45)	0.005	2.29 (1.12–4.68)	0.025

Serum vitamin D (≤30, >30 nmol/L) and MDS (≤2, >2) were analyzed as categorical variables; MDS, magnesium depletion score; OR, odds ratio; CI, confidence interval; Crude model*, included variables MDS, serum vitamin D, and interaction term “MDS × serum vitamin D”; Model 3, adjusted for age, gender, race, education, and PIR based on crude model*; Model 4, adjusted for age, gender, race, education, PIR, diabetes, hypertension, CVD, CKD, dialysis, BMI, time of venipuncture, and vitamin D intake based on crude model*.

Table 4 presents the relationship between serum vitamin D and retinopathy in different MDS groups. In the MDS ≤2 group, serum vitamin D levels ≤30 nmol/L (vs. >30 nmol/L) increased the odds of retinopathy only in univariable analysis (OR = 1.47, 95%CI: 1.05–2.07). In the MDS >2 group, serum vitamin D levels ≤30 nmol/L (vs. >30 nmol/L) was related to higher odds of retinopathy both in univariable analysis (OR = 3.43, 95%CI: 2.01–5.86) and multivariable analysis (OR = 2.90, 95%CI: 1.16–7.24).

**Table 4:** The relationship between serum vitamin D and retinopathy in different MDS groups analyzed by logistic regression analysis.

Variables	Crude Model		Model 1		Model 2	
	OR (95% CI)	*p*	OR (95% CI)	*p*	OR (95% CI)	*p*
MDS ≤ 2 (*n* = 4,521)						
Serum vitamin D > 30 nmol/L	Ref		Ref		Ref	
Serum vitamin D ≤ 30 nmol/L	1.47 (1.05–2.07)	0.026	1.17 (0.81–1.68)	0.391	1.23 (0.83–1.84)	0.293
MDS > 2 (*n* = 432)						
Serum vitamin D > 30 nmol/L	Ref		Ref		Ref	
Serum vitamin D ≤ 30 nmol/L	3.43 (2.01–5.86)	<0.001	3.42 (1.65–7.11)	0.002	2.90 (1.16–7.24)	0.024

MDS, magnesium depletion score; OR, odds ratio; CI, confidence interval; Ref, reference; Crude model, univariable analysis. Model 1, multivariable analysis that adjusted for age, gender, race, education, and PIR; Model 2, multivariable analysis that adjusted for age, gender, race, education, PIR, diabetes, hypertension, CVD, CKD, dialysis, BMI, time of venipuncture, and vitamin D intake.

Because of the effect of age on retinopathy, we excluded 167 participants aged 80 years and older and used data from the remaining 4,786 participants for sensitivity analysis. The results demonstrated that there was still an interaction between MDS and vitamin D on the increased odds of retinopathy [model 4: (OR = 2.48, 95%CI: 1.22–5.05), *P*_interaction_ = 0.014] (Supplementary Table S3). Due to too much missing data for the variable dialysis and the importance of the effect of dialysis on magnesium levels, we performed a sensitivity analysis after excluding the variable dialysis (Supplementary Table S4). The results showed that there was still an interaction between MDS and vitamin D on the risk of retinopathy after the exclusion of the variable dialysis [model 4: (OR = 2.26, 95%CI: 1.11–4.58), *P*_interaction_ = 0.025].

### Moderating effect of MDS in different subgroups

The moderating effect of MDS on the association between serum vitamin D and retinopathy in different subgroups were presented in Table 5. In different subgroups, the interaction between MDS and serum vitamin D on retinopathy was observed in males (OR = 6.88, 95%CI: 1.41–33.66, *P*_interaction_ = 0.019), people with diabetes (OR = 3.43, 95%CI: 1.78–6.63, *P*_interaction_ < 0.001), and people with BMI ≥25 kg/m^2^ (OR = 2.46, 95%CI: 1.11–5.44, *P*_interaction_ = 0.028). In addition, there may be an interaction between MDS and serum vitamin D on retinopathy in people older than 60 years (OR = 2.13, 95%CI: 0.97–4.66, *P*_interaction_ = 0.059).

**Table 5:** Interaction between MDS and serum vitamin D on retinopathy in different subgroups analyzed by logistic regression analysis.

Variables	Model			
	OR (95%CI)	*p*	OR (95%CI)	*p*
	Age < 60 years (*n* = 2,480)		Age ≥ 60 years (*n* = 2,473)	
Serum Vitamin D	1.03 (0.56–1.90)	0.912	1.28 (0.68–2.41)	0.427
MDS	1.88 (0.52–6.88)	0.326	0.54 (0.35–0.85)	0.008
MDS × serum vitamin D	1.12 (0.09–13.52)	0.926	2.13 (0.97–4.66)	0.059
	Male (*n* = 2,482)		Female (*n* = 2,471)	
Serum Vitamin D	0.75 (0.43–1.29)	0.290	1.34 (0.76–2.38)	0.303
MDS	0.87 (0.32–2.38)	0.784	0.66 (0.36–1.21)	0.174
MDS × serum vitamin D	6.88 (1.41–33.66)	0.019	1.20 (0.44–3.28)	0.709
	Diabetes-no (*n* = 3,821)		Diabetes-yes (*n* = 1,132)	
Serum Vitamin D	1.20 (0.72–1.99)	0.469	0.88 (0.48–1.59)	0.654
MDS	0.99 (0.43–2.29)	0.986	0.58 (0.34–0.99)	0.048
MDS × serum vitamin D	1.84 (0.44–7.66)	0.390	3.43 (1.78–6.63)	<0.001
	BMI < 25 kg/m^2^ (*n* = 1,276)		BMI ≥ 25 kg/m^2^ (*n* = 3,677)	
Serum Vitamin D	1.58 (0.67–3.70)	0.283	0.97 (0.62–1.52)	0.904
MDS	0.39 (0.12–1.26)	0.111	0.83 (0.48–1.44)	0.495
MDS × serum vitamin D	1.68 (0.11–26.86)	0.705	2.46 (1.11–5.44)	0.028

Serum vitamin D (≤30, >30 nmol/L) and MDS (≤2, >2) were analyzed as categorical variables; MDS, magnesium depletion score; OR, odds ratio; CI, confidence interval; Model, included variables MDS, serum vitamin D, and interaction term “MDS × serum vitamin D” and adjusted for age, gender, race, education, PIR, diabetes, hypertension, CVD, CKD, dialysis, BMI, time of venipuncture, and vitamin D intake (corresponding subgroup variables are not adjusted in this subgroup analysis).

Among the different MDS groups, only serum vitamin D levels ≤30 nmol/L (vs. >30 nmol/L) were observed to increase the odds of retinopathy in males (OR = 14.07, 95%CI: 1.61–123.16), people with diabetes (OR = 3.71, 95%CI: 1.99–6.94), people older than 60 years (OR = 3.35, 95%CI: 1.37–8.17), and people with BMI ≥25 kg/m^2^ (OR = 2.88, 95%CI: 1.07–7.76) in the MDS >2 group, but not in the MDS ≤2 group (*p* > 0.05) (Supplementary Table S5).

## Discussion

This study examined the relationship between magnesium status and serum vitamin D levels and retinopathy in people aged 40 years and older. Serum vitamin D levels ≤30 nmol/L and high MDS (magnesium deficiency) were associated with higher odds of retinopathy. Moreover, MDS plays a moderating role in the relationship between serum vitamin D and retinopathy, and the moderating effect of MDS was observed only in males, people with diabetes, people older than 60 years, and people with BMI ≥25 kg/m^2^.

Retinopathy is caused by microangiopathy involving small pre-capillary retinal arterioles, capillaries, and small veins (26). Injury is caused by microvascular leakage and microvascular occlusion resulting from rupture of the blood-retinal barrier (26). Several studies have reported the protective role of vitamin D in the development of retinopathy (4, 5, 27). Vitamin D may exert retinal protective effects through antioxidant, anti-inflammatory, anti-angiogenic, and immunomodulatory mechanisms (5). Vitamin D deficiency has been found to be associated with many eye diseases, such as myopia, age-related macular degeneration, glaucoma, diabetic retinopathy, and dry eye (5). Vitamin D has antioxidant and anti-inflammatory properties and plays a role in anti-angiogenesis, regulation of cell proliferation, differentiation, and apoptosis (28, 29). In addition, vitamin D prevents oxidative stress and inflammation in human retinal cells and increases the cellular viability of retinal pigment epithelial cells and various tissues (4). This current study analyzed the relationship between serum vitamin D levels and magnesium status and retinopathy. Our results demonstrated that low vitamin D levels and high MDS were related to higher odds of retinopathy. In addition, MDS plays a moderating role in the effect of serum vitamin D on retinopathy. The risk of retinopathy changed insignificantly with decreasing serum vitamin D levels in the low magnesium depletion group, whereas the risk of retinopathy showed a rapid increase with decreasing serum vitamin D levels in the high magnesium depletion group.

Magnesium plays an important role in maintaining normal metabolism and ionic balance in ocular tissues (30). Membrane-associated ATPases, enzymes for ATP production and hydrolysis are magnesium-dependent (31). In the presence of magnesium deficiency, insufficient activity of antioxidant enzymes leads to lipid peroxidation of polyunsaturated fatty acid-rich membranes by free radicals, thereby impairing retinal function (31). For diabetic retinopathy, insulin resistance decreases intestinal and renal tubular epithelial activity and reduces magnesium absorption by the intestinal and renal epithelium, resulting in low serum magnesium (32). Low serum magnesium levels can further exacerbate insulin resistance, and the two affect each other (33). Magnesium intake can reduce oxidative stress and improve insulin and glucose metabolism (34, 35). In our further analyses, the moderating effect of MDS on the relationship between serum vitamin D and retinopathy was observed only in males, people with diabetes, people older than 60 years, and people with BMI ≥25 kg/m^2^. Sex differences in the moderating effect of MDS may be related to sex hormones. Serum magnesium concentrations have been reported to be positively correlated with estradiol (36). The moderating effect of MDS was significant in people with diabetes and people with BMI ≥25 kg/m^2^ may be associated with insulin and glucose metabolism due to the role of magnesium in insulin and glucose metabolism (34, 35). Since magnesium status plays a moderating role in the effect of serum vitamin D on retinopathy, the corresponding mechanism of effect may need to be further explored.

This study is the first to examine the interaction of magnesium and vitamin D status on the risk of retinopathy in the middle-aged and elderly population based on data from a large nationally representative sample. This study provides epidemiologic evidence for the effect of magnesium modulating vitamin D levels on retinopathy. However, some limitations of this study should be noted. First, this was a cross-sectional study that could not infer causality, and residual confounders may have biased the results. Second, the effects of MDS and vitamin D levels on different subtypes of retinopathy could not be assessed because of the lack of appropriate data. Third, some of the information, such as medical history and physical activity, was obtained through self-report, which may have information bias.

## Conclusion

This study explored the joint effect of magnesium status and serum vitamin D levels on retinopathy in people aged 40 years and older. Magnesium levels may play a moderating role in the relationship between vitamin D and retinopathy. The protective effect of vitamin D against retinopathy was primarily present among those with inadequate magnesium levels. The mechanisms underlying the moderating effect of magnesium status on the relationship between vitamin D and retinopathy may need to be further explored.

## Data availability statement

The original contributions presented in the study are included in the article/Supplementary materials, further inquiries can be directed to the corresponding author/s.

## Ethics statement

The requirement of ethical approval was waived by First Affiliated Hospital of Gannan Medical University, for the studies involving humans because First Affiliated Hospital of Gannan Medical University. The studies were conducted in accordance with the local legislation and institutional requirements. The participants provided their written informed consent to participate in this study.

## Author contributions

LeX: Conceptualization, Funding acquisition, Project administration, Supervision, Writing – original draft, Writing – review & editing. PY: Data curation, Formal analysis, Investigation, Methodology, Writing – review & editing. WL: Data curation, Formal analysis, Investigation, Methodology, Writing – review & editing. LL: Data curation, Formal analysis, Investigation, Methodology, Writing – review & editing. XL: Data curation, Formal analysis, Investigation, Methodology, Writing – review & editing. LiX: Conceptualization, Project administration, Writing – review & editing.

## Footnotes

^1^ https://www.cdc.gov/nchs/nhanes/index.htm
