# Supplementary material for: Magnesium status modulating the effect of serum vitamin D levels on retinopathy: National Health and Nutrition Examination Survey 2005 to 2008
Source: Front Nutr. 2024 Jun 4;11:1408497. doi: 10.3389/fnut.2024.1408497 (PMC11183295; doi:10.3389/fnut.2024.1408497)
Supplement: Supplementary file 1 [file Table_1.DOCX]

**Supplement Table 1**. Difference analysis before and after interpolation of missing values.

| **Variables** | **Before interpolation** | **After interpolation** | **Statistics** | ***P*** |
| --- | --- | --- | --- | --- |
| Education, n (%) |  |  | χ^2^=0.96 | 0.328 |
| Less than high school | 1426 (17.36) | 1426 (17.36) |  |  |
| More than high school | 3526 (82.64) | 3527 (82.64) |  |  |
| Marital status, n (%) |  |  | χ^2^=1.38 | 0.502 |
| Married | 3006 (65.62) | 3007 (65.60) |  |  |
| Never married | 337 (6.11) | 337 (6.11) |  |  |
| Others | 1608 (28.27) | 1609 (28.29) |  |  |
| PIR, n (%) |  |  | χ^2^=1.06 | 0.589 |
| ≤1.3 | 1130 (14.38) | 1221 (14.50) |  |  |
| 1.3-3.5 | 1778 (33.93) | 1906 (33.97) |  |  |
| >3.5 | 1726 (51.69) | 1826 (51.53) |  |  |
| Smoke, n (%) |  |  | χ^2^=0.03 | 0.864 |
| No | 2361 (48.61) | 2362 (48.61) |  |  |
| Yes | 2590 (51.39) | 2591 (51.39) |  |  |
| BMI, kg/m^2^, Mean (S.E) | 29.09 (0.15) | 29.09 (0.15) | t=0.16 | 0.878 |
| CKD, n (%) |  |  | χ^2^=2.63 | 0.105 |
| No | 4009 (86.38) | 4025 (86.29) |  |  |
| Yes | 918 (13.62) | 928 (13.71) |  |  |
| Vitamin A intake, mcg, Mean (S.E) | 638.47 (11.78) | 637.52 (11.63) | t=1.06 | 0.297 |
| HEI-2015, Mean (S.E) | 51.45 (0.43) | 51.40 (0.43) | t=1.71 | 0.097 |
| Magnesium intake, mg, Mean (S.E) | 320.85 (6.95) | 320.44 (6.81) | t=1.23 | 0.229 |
| Total energy, kcal, Mean (S.E) | 2096.35 (21.54) | 2097.52 (21.29) | t=-0.56 | 0.578 |

Notes: PIR, family poverty-to-income ratio; CKD, chronic kidney disease; BMI, body mass index; HEI-2015, Healthy Eating Index-2015.

**Supplement Table 2**. Univariable logistic regression analysis of retinopathy-related covariates.

| **Variables** | **OR (95%CI)** | ***P*** |
| --- | --- | --- |
| Age |  |  |
| <60 years | Ref |  |
| ≥60 years | 1.63 (1.30-2.06) | <0.001 |
| Gender |  |  |
| Male | Ref |  |
| Female | 0.67 (0.57-0.79) | <0.001 |
| Race |  |  |
| Non-Hispanic White | Ref |  |
| Non-Hispanic Black | 1.98 (1.52-2.57) | <0.001 |
| Others | 1.48 (1.13-1.95) | 0.006 |
| Education |  |  |
| Less than high school | Ref |  |
| More than high school | 0.62 (0.50-0.77) | <0.001 |
| Marital status |  |  |
| Married | Ref |  |
| Never married | 1.21 (0.75-1.96) | 0.417 |
| Others | 0.95 (0.76-1.18) | 0.630 |
| PIR |  |  |
| ≤1.3 | Ref |  |
| 1.3-3.5 | 1.25 (0.98-1.59) | 0.073 |
| >3.5 | 0.67 (0.47-0.95) | 0.028 |
| Physical activity |  |  |
| <450 met*minutes/week | Ref |  |
| ≥450 met*minutes/week | 0.93 (0.61-1.43) | 0.739 |
| Unknown | 1.45 (1.00-2.12) | 0.052 |
| Smoke |  |  |
| No | Ref |  |
| Yes | 1.05 (0.82-1.33) | 0.710 |
| Diabetes |  |  |
| No | Ref |  |
| Yes | 4.61 (3.67-5.78) | <0.001 |
| Hypertension |  |  |
| No | Ref |  |
| Yes | 1.70 (1.36-2.13) | <0.001 |
| Dyslipidemia |  |  |
| No | Ref |  |
| Yes | 1.07 (0.74-1.55) | 0.716 |
| CVD |  |  |
| No | Ref |  |
| Yes | 2.16 (1.81-2.57) | <0.001 |
| CKD |  |  |
| No | Ref |  |
| Yes | 2.52 (1.94-3.28) | <0.001 |
| Dialysis |  |  |
| No | Ref |  |
| Yes | 8.63 (1.54-48.48) | 0.016 |
| Unknown | 0.52 (0.32-0.85) | 0.010 |
| BMI |  |  |
| BMI<25kg/m^2^ | Ref |  |
| BMI≥25kg/m^2^ | 1.68 (1.30-2.17) | <0.001 |
| Time of venipuncture |  |  |
| Morning | Ref |  |
| Afternoon | 0.83 (0.64-1.07) | 0.148 |
| Evening | 0.60 (0.40-0.90) | 0.015 |
| Season of sample collection |  |  |
| November 1 through April | Ref |  |
| May 1 through October | 0.90 (0.72-1.14) | 0.373 |
| Vitamin A intake | 1.00 (1.00-1.00) | 0.741 |
| Vitamin D intake |  |  |
| Adequate | Ref |  |
| Inadequate | 1.39 (1.04-1.86) | 0.027 |
| Unknown | 1.29 (0.90-1.85) | 0.164 |
| HEI-2015 | 0.99 (0.99-1.00) | 0.158 |
| Magnesium intake | 1.00 (1.00-1.00) | 0.647 |
| Total energy | 1.00 (1.00-1.00) | 0.920 |

Notes: PIR, family poverty-to-income ratio; CVD, cardiovascular disease; CKD, chronic kidney disease; BMI, body mass index; HEI-2015, Healthy Eating Index-2015; OR, odds ratio; CI, confidence interval.

**Supplement Table 3**. Interaction between MDS and serum vitamin D on retinopathy after exclusion of people aged 80 years and older.

| **Variables** | **Crude Model*** | | **Model 3** | | **Model 4** | |
| --- | --- | --- | --- | --- | --- | --- |
|  | **OR (95% CI)** | ***P*** | **OR (95% CI)** | ***P*** | **OR (95% CI)** | ***P*** |
| Serum vitamin D | 1.50 (1.06-2.11) | 0.023 | 1.17 (0.83-1.64) | 0.358 | 1.07 (0.72-1.57) | 0.739 |
| MDS | 1.59 (0.97-2.62) | 0.063 | 1.31 (0.80-2.14) | 0.280 | 0.81 (0.49-1.36) | 0.422 |
| MDS × serum vitamin D | 2.55 (1.30-4.97) | 0.008 | 3.04 (1.57-5.89) | 0.002 | 2.48 (1.22-5.05) | 0.014 |

Notes: serum vitamin D (≤30, >30 nmol/L) and MDS (≤2, >2) were analyzed as categorical variables; MDS, magnesium depletion score; OR, odds ratio; CI, confidence interval;

Crude model*, included variables MDS, serum vitamin D, and interaction term “MDS × serum vitamin D”;

Model 3, adjusted for age, gender, race, education, and PIR based on crude model*;

Model 4, adjusted for age, gender, race, education, PIR, diabetes, hypertension, CVD, CKD, dialysis, BMI, time of venipuncture, and vitamin D intake based on crude model*.

**Supplement Table 4**. Interaction between MDS and serum vitamin D on retinopathy after exclusion of variable dialysis.

| **Variables** | **Crude Model*** | | **Model 4** | |
| --- | --- | --- | --- | --- |
|  | **OR (95% CI)** | ***P*** | **OR (95% CI)** | ***P*** |
| Serum vitamin D | 1.47 (1.05-2.07) | 0.026 | 1.06 (0.72-1.56) | 0.762 |
| MDS | 1.56 (1.00-2.44) | 0.052 | 0.77 (0.48-1.24) | 0.271 |
| MDS × serum vitamin D | 2.33 (1.17-4.64) | 0.018 | 2.26 (1.11-4.58) | 0.025 |

Notes: serum vitamin D (≤30, >30 nmol/L) and MDS (≤2, >2) were analyzed as categorical variables; MDS, magnesium depletion score; OR, odds ratio; CI, confidence interval;

Crude model*, included variables MDS, serum vitamin D, and interaction term “MDS × serum vitamin D”;

Model 4, adjusted for age, gender, race, education, PIR, diabetes, hypertension, CVD, CKD, BMI, time of venipuncture, and vitamin D intake based on crude model*.

**Supplement Table 5**. The relationship between serum vitamin D and retinopathy in different subgroups.

| **Subgroups** | **MDS≤2** | | **MDS>2** | |
| --- | --- | --- | --- | --- |
|  | **OR (95%CI)** | ***P*** | **OR (95%CI)** | ***P*** |
| Age≥60 | 1.31 (0.69-2.51) | 0.401 | 3.35 (1.37-8.17) | 0.010 |
| Gender-Male | 0.74 (0.41-1.32) | 0.292 | 14.07 (1.61-123.16) | 0.019 |
| Diabetes-Yes | 0.85 (0.48-1.52) | 0.582 | 3.71 (1.99-6.94) | <0.001 |
| BMI≥25kg/m^2^ | 1.19 (0.74-1.92) | 0.454 | 2.88 (1.07-7.76) | 0.037 |

Notes: MDS, magnesium depletion score; OR, odds ratio; CI, confidence interval;

Multivariable analysis adjusted for age, gender, race, education, PIR, diabetes, hypertension, CVD, CKD, dialysis, BMI, time of venipuncture, and vitamin D intake (corresponding subgroup variables are not adjusted in this subgroup analysis).
